# Delayed Diagnoses: Nonspecific Findings and Diagnostic Challenges in Eating Disorders

**DOI:** 10.1155/2009/841037

**Published:** 2009-08-09

**Authors:** Dan Schwarz, Kathryn L. Ponder, Edward R. Feller

**Affiliations:** ^1^Alpert School of Medicine, Brown University, Providence, RI, USA; ^2^Harvard School of Public Health, Harvard University, Boston, MA, USA; ^3^Department of Internal Medicine, Alpert School of Medicine, Brown University, Providence, RI, USA

## Abstract

*Objective*. Eating disorders commonly present with nonspecific findings, masquerading as other, more common etiologies of malnutrition and wasting. In low-prevalence populations, these ambiguities can complicate clinicians' diagnostic reasoning, resulting in delayed or missed diagnoses. *Method*. We report the atypical case of a 51-year-old male with a five-year history of unexplained weight loss despite extensive past medical evaluation. Previous documentation of profound lymphopenia and bone marrow atrophy had not been linked to a known association with eating disorders. *Results*. Evaluation for medical etiologies of wasting was negative. Following psychiatric evaluation, the patient was diagnosed with an eating disorder, not otherwise specified, and admitted to a specialized nutritional rehabilitation program. *Conclusion*. The nonspecific clinical history, physical exam, and laboratory abnormalities of eating disorders can make these diagnoses challenging and delay appropriate treatment. Clinicians should consider eating disorders in patients with malnutrition, severe lymphopenias, and gelatinous marrow transformation early in their workup, so as to avoid potentially negative outcomes.

## 1. Introduction

Eating disorders currently affect five to ten million American females and as many as one million American males [1]. Clinical presentation may be nonspecific, mimicking the signs and symptoms of other common medical etiologies of malnourishment. This is especially true in males and even more so with middle-aged males in whom the diagnoses of eating disorders may not be considered [2]. We report the case of an atypical eating disorder in a 51-year-old male with a long-standing history of progressive wasting and unusual severe hematologic abnormalities.

## 2. Case Report

A 51-year-old male, with a five-year history of a progressive unexplained 50 pound weight loss, presented with one week of worsening generalized weakness and diminished ability to perform activities of daily living. Physical exam was notable for a temperature of 35.9°C with orthostatic hypotension. He was cachectic with extensive muscle wasting, kyphosis, and 2+ bilateral pitting edema in the lower extremities. BMI was 14.8 kg/m^2^.

His medical history was remarkable for prior extensive negative evaluations for infection, neoplasm, malabsorption, or metabolic disease. He had received care from his primary medical doctor as well as a gastroenterologist, endocrinologist, hematologist, and several complementary and alternative care providers. Laboratory results, imaging studies, and multiple GI endoscopies had not demonstrated a medical cause for the patient's persistent malnourishment and continued weight loss. Bone marrow biopsy four years earlier was remarkable for a hypocellular marrow with gelatinous transformation. Complete blood count at that time revealed a normocytic anemia (Hgb: 11.1 g/dl, MCV: 97.9 fL), leukopenia (2.9 × 10^9^/L), and severe lymphopenia (0.6 × 10^9^/L). Two DEXA bone scans (in 2005 and 2006) demonstrated worsening osteoporosis, with lumbar T-scores of −3.4 and −3.6, and femoral T-scores of −3.4 and −3.6, respectively.

At the current admission, blood work was notable for a normocytic anemia (Hgb: 12.0 g/dl, MCV: 94.8 fL), as well as a mildly increased white blood cell count (11.9 × 10^9^/L) with profound lymphopenia (0.0 × 10^9^/L) and 45% band neutrophils. Platelets were 160 × 10^9^/L. Reticulocyte index was 0.36 and iron studies were consistent with anemia of chronic disease (iron: 71 ug/dl, ferritin: 676 ng/mL, TIBC: 151 ug/dl, transferrin saturation: 47%). The patient was hypovolemic (BUN/Cr: 72.0) and hyponatremic (Na: 122 mEq/L) with low serum protein (5.4 g/dl), albumin (2.6 g/dl), and prealbumin (7.8 mg/dl). TSH was normal, aminotransferases were mildly elevated, and HIV and viral hepatitis serologies were negative.

Psychiatric evaluation, which had not been previously done, revealed a cognitively-intact man with poor insight and judgment regarding his condition. Depressive symptoms were noted, but he denied any history of suicidal ideation or self-injurious behaviors. No signs of psychosis were found. Ritualistic food preparation and restricting dietary habits were noted, although he did not endorse typical anorexia nervosa obsessions about body image or weight, nor did these appear to be consistent with obsessive-compulsive disorder. He expressed feelings of guilt and confusion with regards to sexual orientation and religious beliefs.

Following extensive negative testing for other etiologies of cachexia, the patient was diagnosed with an eating disorder, not otherwise specified (ED-NOS) and a mood disorder, NOS. He was placed in a skilled nursing facility for physical rehabilitation, pending placement in a day hospital program for eating disorder-related psychotherapy. 

## 3. Discussion

Despite a five year history of weight loss and extensive medical evaluations, our patient had no prior psychiatric evaluation. The nonspecific and diverse clinical histories, physical exam findings, and laboratory workup of eating disorders are often inappropriately attributed to other, more common causes of malnourishment and wasting.

Eating disorder diagnoses in males may be particularly challenging due to the low prevalence of the disorders in this population. While men may account for up to 25% of binge eating disorder diagnoses, they only account for approximately 10% of AN or bulimia nervosa diagnoses [3]. Our patient's age also placed him in a low prevalence group. Though few epidemiological studies exist, data suggests that the mean age of onset of eating disorders in males is similar to that in females [4], most commonly between adolescence and the mid 20 s [5–8]. One study found that only 5% of males (2/42) and 2% of females (12/557) had onset of AN after the age of 30 [9]. In light of these data, our patient's presentation is remarkable for his respective age and sex demographics, clearly placing him in a minority among other patients with similar diagnoses.

Delayed diagnosis and treatment may be more common in males with non-AN eating disorders compared to those with AN. One study of 135 men with eating disorders found that men with AN exhibited a mean delay in treatment of only two years, while men with non-AN eating disorders had a mean delay of approximately eight years [6]. Our patient's diagnosis of an ED-NOS and his delay in diagnosis and treatment of over five years are consistent with these data.

Hematologic abnormalities may be an important clue to an underlying eating disorder, although anemia, leukopenia, severe lymphopenia, and bone marrow dysfunction occur in other cachectic states as well [5, 7, 8, 10]. A recent review of patients with anorexia nervosa (AN) documented approximately one third who were anemic, most commonly with a normocytic anemia. Leukopenias were present in 29–36% of patients across five studies, and lymphopenias in a further three studies [7]. Our patient's severe lymphopenia (absolute lymphocyte count: 0.0 × 10^9^/L) is remarkable, even in the context of eating disorders: of these three studies that documented lymphopenias in AN patients, only one documented lymphopenias that were statistically significant when compared to controls groups, and none of the studies showed mean absolute lymphocyte counts less than 1.3 × 10^9^/L [10–12].

Bone marrow atrophy and subsequent dysfunction are the likely etiology of these hematologic findings. Gelatinous marrow transformation (GMT), commonly found in malnourished states such as AIDS, alcoholism, malabsorptive diseases, and malignancies [13], has also been documented in patients with eating disorders. In a study of AN patients' bone marrow, GMT was documented in 50% (22/44) of the patients. The pattern of bone marrow degeneration correlated with the degree of weight loss, and higher rates of peripheral cytopenias occurred in patients with gelatinous transformation [5]. Given these findings, the extreme anemia, leukopenia, and lymphopenia seen in this patient are likely secondary to the long-standing hypoplastic gelatinous transformation of his marrow. The patient's low reticulocyte index (0.36) further supports this theory.

## 4. Conclusions

In severe malnutrition, nondiagnostic hematologic abnormalities are often present. We have reported a case in which, secondary to an eating disorder, a middle-aged male demonstrates many of these findings. This case demonstrates that the diagnoses of eating disorders can be challenging, especially in low-prevalence populations such as our patient, a middle-aged male. Marked weight loss in conjunction with cytopenias or marrow atrophy should suggest the possibility of an eating disorder in select undiagnosed patients.

## References

[1] Patrick L (2002). Eating disorders: a review of the literature with emphasis on medical complications and clinical nutrition. *Alternative Medicine Review*.

[2] Siegel JH, Hardoff D, Golden NH, Shenker IR (1995). Medical complications in male adolescents with anorexia nervosa. *Journal of Adolescent Health*.

[3] Weltzin TE, Weisensel N, Franczyk D, Burnett K, Klitz C, Bean P (2005). Eating disorders in men: update. *Journal of Men's Health and Gender*.

[4] Woodside DB, Garfinkel PE, Lin E (2001). Comparisons of men with full or partial eating disorders, men without eating disorders, and women with eating disorders in the community. *American Journal of Psychiatry*.

[5] Abella E, Feliu E, Granada I (2002). Bone marrow changes in anorexia nervosa are correlated with the amount of weight loss and not with other clinical findings. *American Journal of Clinical Pathology*.

[6] Carlat D, Camargo C, Herzog D (1997). Eating disorders in males: a report on 135 patients. *American Journal of Psychiatry*.

[7] Hutter G, Ganepola S, Hofmann W-K (2009). The hematology of anorexia nervosa. *International Journal of Eating Disorders*.

[8] Miller KK, Grinspoon SK, Ciampa J, Hier J, Herzog D, Klibanski A (2005). Medical findings in outpatients with anorexia nervosa. *Archives of Internal Medicine*.

[9] Joughin NA, Crisp AH, Gowers SG, Bhat AV (1991). The clinical features of late onset anorexia nervosa. *Postgraduate Medical Journal*.

[10] Devuyst O, Lambert M, Rodhain J, Lefebvre C, Coche E (1993). Haematological changes and infectious complications in anorexia nervosa: a case-control study. *Quarterly Journal of Medicine*.

[11] Lambert M, Hubert C, Depresseux G (1997). Hematological changes in anorexia nervosa are correlated with total body fat mass depletion. *International Journal of Eating Disorders*.

[12] Nagata T, Kiriike N, Tobitani W, Kawarada Y, Matsunaga H, Yamagami S (1999). Lymphocyte subset, lymphocyte proliferative response, and soluble interleukin-2 receptor in anorexic patients. *Biological Psychiatry*.

[13] Böhm J (2000). Gelatinous transformation of the bone marrow: the spectrum of underlying diseases. *American Journal of Surgical Pathology*.

